# Phenotypic and metabolomic characteristics of mouse models of metabolic associated steatohepatitis

**DOI:** 10.1186/s40364-023-00555-9

**Published:** 2024-01-09

**Authors:** Cian-Ru Yang, Wen-Jen Lin, Pei-Chun Shen, Pei-Yin Liao, Yuan-Chang Dai, Yao-Ching Hung, Hsueh‐Chou Lai, Shiraz Mehmood, Wei-Chung Cheng, Wen-Lung Ma

**Affiliations:** 1 Program for Health Science and Industry, Graduate Institute of Biomedical Sciences, and Department of Medicine, and Tumor Biology Center, School of Medicine, China Medical University, Taichung, Taiwan; 2 Department of Medical Research, Department of Gynecology and Obstetrics, and Department of Gastroenterology, China Medical University Hospital, Taichung, Taiwan; 3 Department of Pathology, Ditmanson Medical Foundation Chia-Yi Christian Hospital, Chia-Yi City, Taiwan; 4 Department of Gynecology and Obstetrics, Asia University Hospital, Taichung, Taiwan; 5 Graduate Institute of Integrated Medicine, College of Chinese Medicine, China Medical University, Taichung, Taiwan; 6 Center for Digestive Medicine, Department of Internal Medicine, China Medical University Hospital, Taichung, Taiwan

**Keywords:** Metabolic associated steatohepatitis, Metabolome, Lipoprotein

## Abstract

**Background:**

Metabolic associated steatohepatitis (MASH) is metabolic disease that may progress to cirrhosis and hepatocellular carcinoma. Mouse models of diet-induced MASH, which is characterized by the high levels of fats, sugars, and cholesterol in diets, are commonly used in research. However, mouse models accurately reflecting the progression of MASH in humans remain to be established. Studies have explored the potential use of serological metabolites as biomarkers of MASH severity in relation to human MASH.

**Methods:**

We performed a comparative analysis of three mouse models of diet-induced MASH in terms of phenotypic and metabolomic characteristics; MASH was induced using different diets: a high-fat diet; a Western diet; and a high-fat, high-cholesterol diet. Liver cirrhosis was diagnosed using standard clinical approaches (e.g., METAVIR score, hyaluronan level, and collagen deposition level). Mouse serum samples were subjected to nuclear magnetic resonance spectroscopy–based metabolomic profiling followed by bioinformatic analyses. Metabolomic analysis of a retrospective cohort of patients with hepatocellular carcinoma was performed; the corresponding cirrhosis scores were also evaluated.

**Results:**

Using clinically relevant quantitative diagnostic methods, the severity of MASH was evaluated. Regarding metabolomics, the number of lipoprotein metabolites increased with both diet and MASH progression. Notably, the levels of very low-density lipoprotein (VLDL) and low-density lipoprotein (LDL) significantly increased with fibrosis progression. During the development of diet-induced MASH in mice, the strongest upregulation of expression was noted for VLDL receptor. Metabolomic analysis of a retrospective cohort of patients with cirrhosis indicated lipoproteins (e.g., VLDL and LDL) as predominant biomarkers of cirrhosis.

**Conclusions:**

Our findings provide insight into the pathophysiology and metabolomics of experimental MASH and its relevance to human MASH. The observed upregulation of lipoprotein expression reveals a feedforward mechanism for MASH development that may be targeted for the development of noninvasive diagnosis.

**Supplementary Information:**

The online version contains supplementary material available at 10.1186/s40364-023-00555-9.

## Background

Metabolic dysfunction-associated steatotic liver disease (MASLD) is formerly known as nonalcoholic fatty liver disease (NAFLD) and is a major cause of chronic liver disease worldwide [1–4]. MASLD can develop as hepatic steatosis or severe conditions involving inflammation, such as metabolic dysfunction-associated steatohepatitis (MASH), liver fibrosis, cirrhosis, hepatocellular carcinoma (HCC), and liver failure [3, 4]. MASH is characterized by hepatic inflammation, hepatocyte ballooning, and intrahepatic fat accumulation [5, 6]. Patients with MASH are at a higher risk of liver cancer, liver failure, and cardiovascular disease than are healthy individuals [2, 5]. Key risk factors for MASH include diabetes, obesity, age, ethnicity, sex, and genetic polymorphisms, which can also affect MASH progression [7, 8]. These risk factors reflect the complex and heterogeneous nature of MASH [9]. Although extensive studies have been conducted on MASLD and MASH, the precise mechanisms underlying the development of MASLD and its progression to MASH have yet to be elucidated.

Several genetic and dietary models of MASH have been developed using various research animals for mimics human MASH [10, 11]. The most commonly used model for studying diet-induced MASLD is the inbred C57BL/6 mouse model [11, 12]. Diets used for inducing MASH include high-fat diets (HFDs) and methionine- and choline-deficient diets [13, 14]. HFD-induce MASLD mimicking the major histopathological and pathogenic features of human MASLD [15, 16]. Although HFD-induced experimental MASLD can mimic the altered metabolic pattern observed in human MASLD, experimental MASLD cannot progress to a severe hepatic pathology. To closely mimic human MASH, animals are sometimes fed an HFD supplemented with other additives, such as fructose and cholesterol [17, 18]. High cholesterol intake can induce dyslipidemia and insulin resistance and is a crucial factor associated with hepatic inflammation and MASH progression in both animal models and humans [15, 19–21]. Fructose accelerates MASH progression through increased inflammation and fibrosis in mice [22–24]. These experimental diets as well as different diet types and feeding lengths induce MASLD/MASH with varying degrees of severity.

Because experimental diet-induced MASH models are developed to mimic the pathophysiology of human MASH, obtaining a detailed account of serological markers is essential. However, few studies have focused on this topic. A comparative analysis of MASH induced by different diets in terms of the changes in serological metabolites is necessary; in addition, the likely association between metabolomic profile and MASH severity must be investigated. Identifying potential metabolic markers and exploring their association with disease progression would be beneficial for early clinical diagnosis of MASLD.

Therefore, we conducted the present study to compare three mouse models of diet-induced MASH in terms of phenotypic and metabolomic characteristics. MASH was induced using different diets: an HFD; a Western diet (WD; high-fat, high-fructose diet); and a high-fat, high-cholesterol diet (HFC). The clinically relevant quantitative diagnosis was performed to evaluate MASH severity in mice. Nuclear magnetic resonance (NMR) spectroscopy–based metabolomic profiling was performed using serum samples obtained from the experimental mice. The acquired data were subjected to bioinformatic analyses to evaluate the MASH severity and phenotypic progression.

## Material & methods

### Animal diets and experimental design

Thirty male C57BL/6 J mice (age, 7 weeks) were purchased from the National Laboratory Animal Center and were housed individually. After 1 week of adaption and quarantine, the mice were divided into three groups on the basis of diet: an HFD group (the mice obtained 60% of their total energy from fat; 58Y1; Young Li Trading Co., Ltd., Taiwan), a WD group (the mice obtained 39.9% of their total energy from fat and 44.1% of their total energy from carbohydrates [fructose]; 5TJN; Young Li Trading Co., Ltd., Taiwan), and an HFC group (the mice obtained 39.4% of their total energy from fat and 2% of their total energy from cholesterol; 5S8X; Young Li Trading Co., Ltd., Taiwan). The mice were fed experimental diets for 16 and 32 weeks to induce MASLD/MASH with varying degrees of severity. At the end of the feeding program, the mice were euthanized, and their liver specimens and blood samples were collected for further analysis. Through cardiac puncture, blood samples were collected into ethylenediaminetetraacetate-coated tubes and subsequently centrifuged at 1000 × *g* for 10 min at 4 °C. The collected liver specimens were cut into thin Sects. (0.5 cm × 0.5 cm), fixed in 10% formalin, and subjected to paraffin embedding. The protocols for the animal experiments were approved by the Animal Ethics Committee of China Medical University (approval number: CMUIACUC-2021–061).

### Real-time quantitative polymerase chain reaction

Total RNA was extracted from the liver tissues of the mice by using TRIzol (T9424, Sigma-Aldrich, St. Louis, Missouri, USA) according to the manufacturer’s instructions [25]. The obtained RNA (5 μg) was reverse-transcribed using the PrimeScript RT Reagent Kit (RR037A; TaKaRa, Tokyo, Japan) according to the manufacturer’s instructions [26]. Quantitative polymerase chain reaction (PCR) was performed using the KAPA SYBR FAST qPCR Master Mix (KM4100; KAPA Biosystems) with specific primers ( Supplementary Table 1); for this, the Azure Cielo Real-Time PCR System (Azure Biosystems, Dublin, CA, USA) was used. Gene expression levels were normalized against the expression level of actin, and the relative changes in gene expression were quantified using the 2^−ΔΔCt^ method.

### Detection of hyaluronan through sandwich enzyme-linked immunosorbent assay

The presence of hyaluronan in the serum samples of the experimental mice was detected through sandwich enzyme-linked immunosorbent assay (ELISA). All blood samples were centrifuged at 1000 × *g* for 15 min at 4 °C; the obtained plasma samples were subjected to sandwich ELISA, which was performed using Quantikine ELISA Kits (DHYAL0; R&D System, Minneapolis, MN, USA) according to the manufacturer’s instructions. In brief, all reagents, working standards, and samples were prepared as directed. To each well, 50 μL of Assay Diluent RD1-14 was added followed by 50 μL of the standard, control, or sample. The ELISA plate was incubated on a horizontal orbital shaker (70 rpm) for 2 h at 25 °C. After incubation, each well was aspirated and washed five times with 400 μL Wash Buffer. Next, 100 μL of hyaluronan conjugate was added to each well. The plate was covered with a clean adhesive strip and incubated on the shaker for 2 h at room temperature. After incubation, the wells were aspirated and washed as indicated. Subsequently, 100 μL of substrate solution was added to each well. The plate was incubated in the dark for 30 min at room temperature. After incubation, 100 μL of stop solution was added to each well. Within 30 min of the addition of the stop solution, absorbance was measured at 450 and 570 nm by using a microplate reader; for background correction, the readings obtained at 570 nm were subtracted from those obtained at 450 nm.

### Histopathological analysis

Paraffin-embedded liver tissue sections were stained with hematoxylin–eosin (H&E) and Masson’s trichrome for histopathological analysis [27]. Using the METAVIR scoring system, a licensed pathologist who was blinded to the group allocation scored the Masson’s trichrome–stained liver tissues for fibrosis and cirrhosis [28–30]. Collagen fibers were detected in the tissue sections stained with Masson’s trichrome. Tissue images were captured at 20 × magnification. The area containing collagen fibers was assessed using ImageJ [31]. Collagen deposition was quantified using 20 images per liver sample.

### NMR spectroscopy–based metabolomic profiling

A retrospective cohort of patients with cirrhosis was analyzed. Serum samples were collected from 80 patients with HCC and subjected to NMR spectroscopy–based metabolomic profiling by using the Nightingale Health platform (Helsinki, Finland) [32]. This platform facilitates the simultaneous detection of 151 serum biomarkers and provides a comprehensive spectrum of metabolites. The biomarkers include lipid metabolites, such as cholesterol, triglycerides, various fatty acids, apolipoprotein (Apo)A1, and ApoB; amino acid; glycolysis-related metabolite; ketone bodies; creatinine; albumin; and glycoprotein acetyls. The size, subclass distribution, and loading lipids of lipoprotein metabolites can also be analyzed using this platform. Details regarding the observed serological metabolites, including their abbreviations and units, are presented in Supplementary Table 2.

For metabolomic profiling, mouse plasma samples were assessed through NMR spectroscopy (Ascend 600C) on the Burker high-throughput metabolomics platform. The standardized platform included Bruker IVDr Lipoprotein Subclass Analysis (model version: PL-5009–01/001) and the automated quantification of small metabolites (model version: Quant-PS 2.0.0). Details regarding the metabolites, including their abbreviations and units, are presented in Supplementary Table 3 and 4.

### Retrospective analysis of patients with fibrosis or cirrhosis

A total of 113 patients who received a confirmed diagnosis of HCC between 2009 and 2013 at China Medical University Hospital were retrospectively included in this study. This retrospective analysis include patient serum samples and liver biopsies. Patients with missing liver biopsy or key hematological data; those with blood samples unsuitable for metabolite quantification; those aged < 18 or > 80 years; those receiving long-term drug therapy; and those with advanced cancer metastasis, HIV infection, autoimmune disease, or other liver-related comorbidities (e.g., Wilson’s disease, haemochromatosis, alpha-1 antitrypsin deficiency, lupoid hepatitis, and cholestatic or vascular liver disorders) were excluded from this study. Finally, 80 patients were included in the metabolomic analysis. The patients’ demographic and medical data were recorded by trained research assistants. The effects of the following covariates were adjusted in the models used for statistical analysis: age (years), sex (male or female), body mass index (kg/m^2^), smoking status (yes [smoked for at least 1 year] or no), hypertension (blood pressure of > 140/90 mmHg or previous physician diagnosis), type 2 diabetes (fasting glucose level of > 126 mg/dL or previous physician diagnosis), hepatitis B surface antigen (yes or no), hepatitis C antibody (yes or no), Child–Pugh score (A + B or C), ascites (yes or no), hepatic steatosis (yes or no), tumor size (cm), cancer stage (I + II or III + IV), microscopic venous invasion (yes or no), macroscopic venous invasion (yes or no), lymph node involvement (yes or no), and capsule (yes or no) and satellite nodules (yes or no). This study was approved by the Ethics Committee of China Medical University. Written informed consent was obtained from all patients.

### Bioinformatic analyses

For metabolite analysis, all data were subjected to normality tests (Shapiro–Wilks and Anderson–Darling tests) and Q–Q plot analysis [33]. Among the metabolites, > 50% were abnormally distributed; therefore, subsequent analyses were performed using nonparametric tests [34]. The metabolites were divided into three groups: lipoproteins and small metabolites, lipoprotein metabolites, and small metabolites.

The metabolite groups were subjected to principal component analysis (PCA) [34, 35]. Hierarchical clustering heatmaps were independently constructed using R package gplots [36, 37]. Before analysis, each feature was scaled to have a mean value of 0 and a standard deviation value of 1.

Multiple comparisons were performed using the Kruskal–Wallis test in different groups of diets [38–40]. Statistical significance was set at *p* < 0.05. To analyze the differentially expressed metabolites, the Wilcoxon rank-sum test was performed, with fold changes and adjusted *p* values calculated for between-group comparisons [41]. Metabolites were considered to have significance when the corresponding *p* values were < 0.05 and log2 fold changes were greater than or equal to 1 or less than or equal to − 1. Spearman correlation analysis was performed to investigate the correlation between collagen scores and metabolites [42]. A *p* value of < 0.05 and a correlation coefficient of > 0.3 indicated statistical significance.

For the cohort of patients with cirrhosis, between-group comparisons of metabolites were performed using a two-tailed Student’s *t* test. The resulting *p* values were adjusted for multiple comparisons by using the Benjamini–Hochberg correction method [43, 44]. Statistical significance was set at a q value of < 0.05. Significant metabolites were selected to construct a Pearson correlation matrix, which was subsequently subjected to hierarchical cluster analysis, in which the Euclidean distance [45] was measured using the average method. All statistical analyses were performed using R (version 4.1.0).

### Statistical analysis

The study data are presented in terms of mean ± standard deviation values and were analyzed with GraphPad Prism (version 8.0). The unpaired Student’s *t* test was used for between-group comparisons. A *p* value of < 0.05 was considered to be significant.

## Results

### Phenotypic characteristics of mouse models of diet-induced MASH

**Fig. 1:**
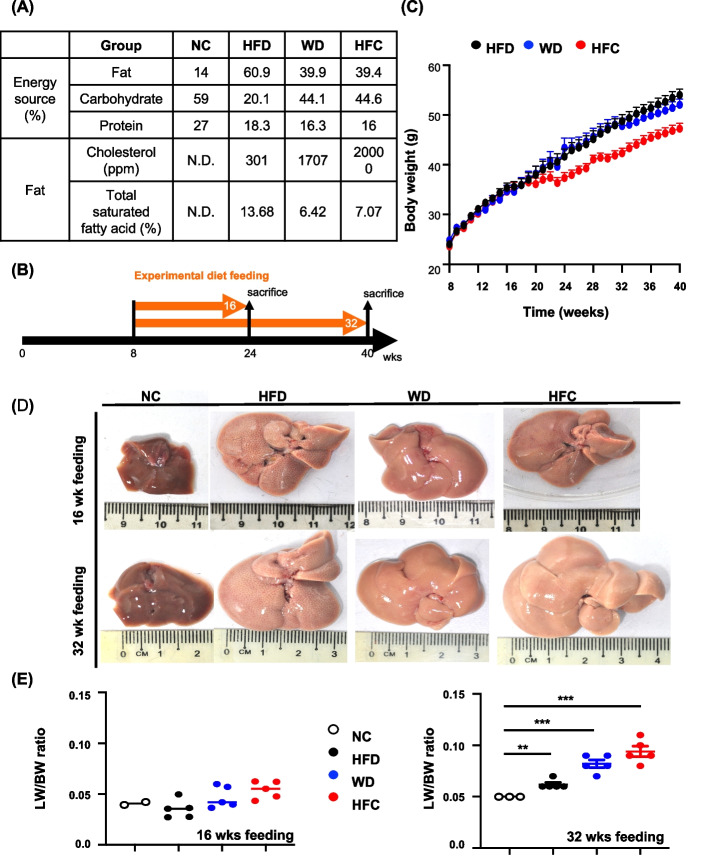
Phenotypic characteristics of mouse models of diet-induced MASH. **A** Nutrients and energy source in the three experimental diets. **B** Experimental design for establishing mouse models of MASLD/MASH. Mice were fed various diets for 16 and 32 weeks and then euthanized; liver specimens and blood samples were collected for analysis. **C** Changes in the body weight of mice after 32 weeks of feeding. **D** Livers of mice receiving different diets fed for 16 and 32 weeks. **E** Liver weight/body weight ratio of mice fed different diets for 16 weeks (left) and 32 weeks (right). Statistical significance: *p < 0.05, **p < 0.01, and ***p < 0.001. NC: normal chow; HFD: high-fat diet; WD: Western diet; HFC: high-fat, high-cholesterol diet; MASH, metabolic dysfunction-associated steatohepatitis; ppm: parts per million

Mice fed a normal chow (NC) diet were used as the control group. Figure 1A presents the nutritional composition of each diet and the percentage of total energy contributed by each dietary component. The feeding schedule is depicted in Fig. 1B. The changes in the body weights (BWs) of the mice in the three experimental diet groups were recorded. A rapid increase was noted in the BW of all three groups; however, measurements performed when the mice were aged 24 weeks revealed that BW gain was faster in the HFD and WD groups than in the HFC group (Fig. 1C). After 16 and 32 weeks, the livers were larger and lighter (in color) in the experimental groups than in the control group (Fig. 1D).

The ratio of liver weight (LW) to BW is a robust and vital indicator of liver homeostasis. Normally, the LW/BW ratio ranges from 4.5% to 5%. Changes in the LW/BW ratio indicate liver under pathological insults. After 16 weeks of feeding, we noted no significant change in the LW/BW ratio in the four (HFD, WD, HFC, and NC) groups. By contrast, after 32 weeks of feeding, the ratio increased significantly in the three experimental groups compared with the ratio in the control group (Fig. 1E). Thus, prolonged intake of the experimental diets led to pathological changes in the mouse liver that accelerated diet-induced MASLD/MASH. In summary, all three experimental diets induced MASLD/MASH with varying degrees of severity.

### Importance of clinically relevant diagnostic methods for the severity of diet-induced MASH in mice

**Fig. 2:**
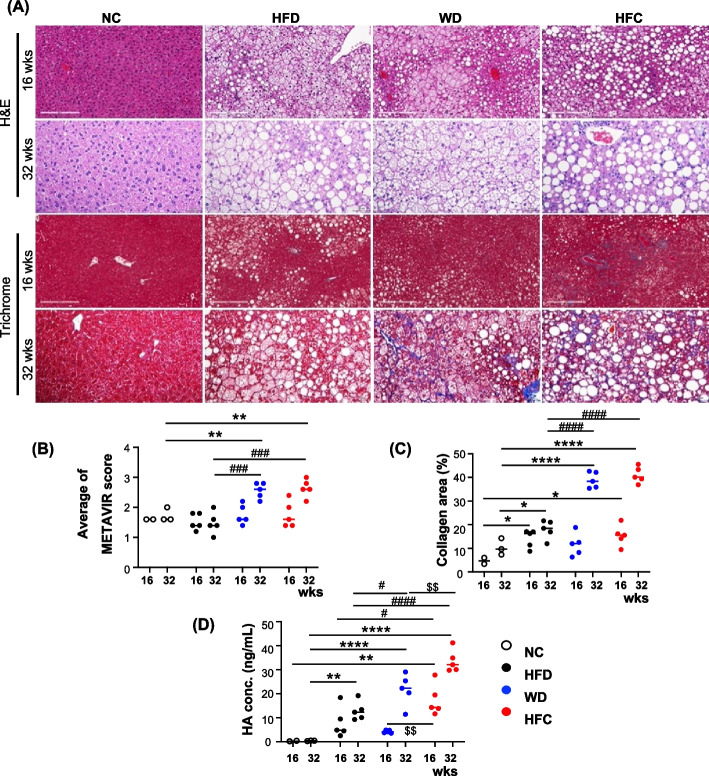
Evaluation of the severity of diet-induced metabolic dysfunction-associated steatohepatitis by using clinically relevant diagnostic methods. **A** Analysis of pathological features through hematoxylin–eosin staining. Hepatocyte ballooning and intrahepatic lipid accumulation (droplets) were observed. The severity of liver fibrosis was evaluated through Masson’s trichrome staining. The stained samples were observed under a bright-field microscope; scale bar = 200 mm. **B** METAVIR scores were quantified by a licensed pathologist who was blinded to the group allocation. **C** Masson’s trichrome staining scores for the observed collagen fibers were quantified using ImageJ. **D** Serum levels of hyaluronan were measured through enzyme-linked immunosorbent assay. Statistical significance: *p < 0.05, ***p < 0.001, and ****p < 0.0001, compared with normal chow; #*p* < 0.05, ###*p* < 0.001, and ####*p* < 0.0001, compared with the high-fat diet; $*p* < 0.05 and $$*p* < 0.01, compared with the Western diet

Clinically relevant quantitative diagnosis was performed to evaluate the severity of diet-induced MASH. The results of histopathological staining performed using the liver tissue samples of each group are presented in Fig. 2A. The results of H&E staining revealed no abnormalities in the control group but gradual pathological changes (e.g., hepatic steatosis and hepatocyte ballooning; after 16 and 32 weeks) in the experimental groups (Fig. 2A). Moreover, Masson’s trichrome staining revealed prominent collagen fibers (liver fibrosis) in the WD and HFC groups (after 16 and 32 weeks; Fig. 2A). The METAVIR scoring system was used to evaluate the degree of liver fibrosis [46]. The METAVIR grading criteria were as follows: F0, no fibrosis; F1, portal fibrosis without septa; F2, portal fibrosis with a few septa; F3, portal fibrosis with numerous septa without cirrhosis; F4, cirrhosis and single-blinded diagnosis by a certified pathologist. No significant difference was found among the four groups with respect to fibrosis scores after 16 weeks of feeding (Fig. 2B). However, after 32 weeks of feeding, the fibrosis scores of the WD and HFC groups were higher than those of the NC and HFD groups; this finding suggests that liver fibrosis was more severe in the WD and HFC groups than in the NC and HFD groups (Fig. 2B). After 16 weeks of feeding, no significant changes were noted in the level of collagen deposition in the four groups. However, after 32 weeks of feeding, the level of collagen deposition increased significantly in the WD and HFC groups compared with the levels in the NC and HFD groups; this finding indicates more severe fibrosis in the WD and HFC groups (Fig. 2C).

In addition to histopathological analysis, this study quantified a clinically used serological biomarker of MASLD/MASH—hyaluronan [46]. The serum level of hyaluronan was measured through sandwich ELISA (Fig. 2D). No significant changes were noted in the level of hyaluronan in the NC group. Nevertheless, the level of hyaluronan increased significantly in the HFD and WD groups; the highest degree of increase was noted in the HFC group. However, the gradual changes observed in the level of hyaluronan in the HFD group were nonsignificant. These findings indicate prolonged intakes of the WD and HFC increased the level of hyaluronan in the blood of the experimental mice (Fig. 2D). In the study of liver fibrosis, α-SMA and collagen I are important markers. Therefore, we selected α-SMA and collagen I for evaluating liver fibrosis. Notably, BW gain was slower and the LW/BW ratio was higher in the HFC group than in the other groups; this indicates a more rapid progression of MASH in the HFC group than in the HFD and WD groups (Fig. 1E). Furthermore, the WD and HFC accelerated MASH progression, leading to advanced, conditions such as liver fibrosis. In summary, the severity of MASLD/MASH induced by the three experimental diets could be quantified and scored using clinically relevant diagnostic methods.

### Lipoprotein metabolites are key biomarkers of diet-induced MASH in mice

**Fig. 3:**
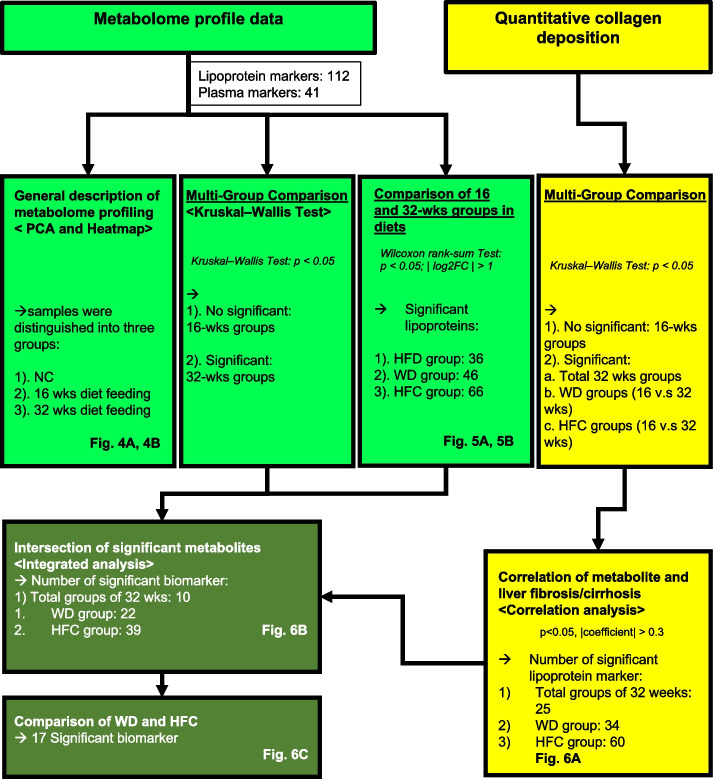
Strategies for metabolomic analysis. Metabolomic profiles (light green) and collagen fiber quantitation data (yellow) were analyzed through differential gene expression and correlation analyses. The correlation of serological metabolites with fibrosis severity was investigated (dark green), and the list of significant metabolites was shortened. Unbiased clustering was performed through primary component analysis and heatmap analysis; the results are presented in Fig. 4A, B. The results of a comparison of the 16- and 32-week feeding durations in terms of metabolomic changes in the experimental mice (time effect) are presented in Fig. 5A, B (Wilcoxon rank-sum test). A multigroup comparison (Kruskal–Wallis test) was performed to differentiate between the significant metabolites. Significant metabolites were compared with significant collagen deposition (Kruskal–Wallis test; yellow squares); the correlation between collagen deposition and metabolome was investigated (correlation analysis; yellow square; Fig. 6A). The intersection of significant metabolites among the 32-week, WD, and HFC groups is depicted in Fig. 6B (dark-colored square), and the shortened metabolite list is presented in Fig. 6C

The MASLD/MASH phenotype induced by experimental diets is quantifiable and scalable; thus, the corresponding serological metabolomic characteristics can be aligned. Mouse serum samples were subjected to NMR spectroscopy–based metabolomic profiling followed by bioinformatics analyses. The analytical logic and diagram are presented in Fig. 3. The data corresponding to serological metabolome and collagen deposition (Masson’s trichrome staining) were discovered to be correlated. A total of 41 biomarkers and 112 lipoprotein metabolites were assessed (Fig. 3). PCA and heatmap analysis were performed to identify the overall pattern and trends of changes in metabolites. Multiple comparisons were performed to determine the significant differences between the groups (significance was observed after 32 weeks of feeding; Kruskal–Walls test) and between the feeding durations (16 and 32 weeks; time effect; Wilcoxon rank-sum test). Additional multiple comparisons were performed for the collagen-related quantitative data (Fig. 3) to determine the differences between the feeding durations; various metabolites were identified in this analysis (Fig. 5A, B). In the case of significant between-group differences in collagen deposition level, correlation and comparative analyses of metabolites and collagen deposition levels were performed. A trimmed list of significant metabolites was obtained (Fig. 6A). Finally, the most significant metabolites (*n* = 17) were selected from the WD and HFC groups (Fig. 6B).

**Fig. 4:**
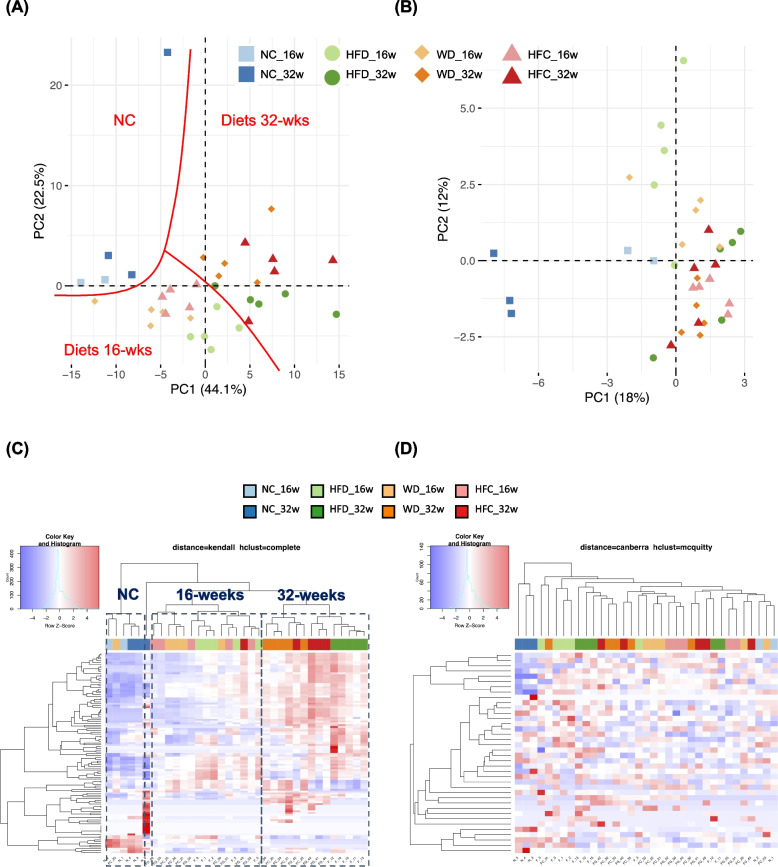
Lipoproteins as predominant metabolites in mouse models of diet-induced metabolic dysfunction-associated steatohepatitis. **A** Plot generated through principal component analysis (PCA) of the lipoproteins of mice fed with NC, HFD, WD, or HFC (16 and 32 weeks). **B** Plot generated through the PCA of the serological metabolites of mice fed with NC, HFD, WD, or HFC (16 and 32 weeks). PC1: principal component 1; PC2: principal component 2. Each point represents the metabolite profile of a biological replicate. **C** Heatmap exhibiting prominent differences in lipoprotein patterns among NC, 16-week experimental diet, and 32-week experimental diet. **D** Heatmap exhibiting no distinct pattern of serological metabolites. NC: normal chow; HFD: high-fat diet; WD: Western diet; and HFC: high-fat, high-cholesterol diet

PCA was performed for the unbiased clustering of the obtained metabolomic data. All metabolites (153 biomarkers), the lipoprotein metabolites alone (112 biomarkers), and the small metabolites alone (41 biomarkers) were assessed. For all metabolites, the first principal component (PC1) accounted for 18.9% of the overall variability, whereas the second principal component (PC2) accounted for 35.3%. The PCA plot based on the top two principal components indicated that all samples could be categorized into NC, 16-week-experimental-diet, and 32-week-experimental-diet groups (Supplementary Fig. 1A). For the lipoprotein metabolites, PC1 accounted for 44.1% of the overall variability, whereas PC2 accounted for 22.5%. The PCA plot indicated that all samples could be categorized into NC, 16-week-experimental-diet, and 32-week-experimental-diet groups (Fig. 4A). For the small metabolites, PC1 accounted for 18% of the overall variability, whereas PC2 accounted for 12%. The PCA plot indicated that the samples could not be categorized (Fig. 4B). The heatmap reveals distinct regions with contrasting colors, indicating significant differences across different variables or conditions. Heatmaps were constructed to visualize the distribution of the metabolites across groups. The heatmap corresponding to all metabolites revealed strong differences among the NC, 16-week-experimental-diet, and 32-week-experimental-diet groups (Supplementary Fig. 1B). The heatmap corresponding to the lipoprotein metabolites revealed strong differences among the NC, 16-week-experimental-diet, and 32-week-experimental-diet groups (Fig. 4C). The heatmaps revealed no difference between the groups in terms of serological biomarkers (Fig. 4D). These findings are consistent with the trends of the corresponding PCA results. In summary, the PCA and heatmap analysis indicated that lipoproteins undergo gradual changes and play major roles in the progression of MASLD/MASH.

### VLDL and LDL are the predominant metabolites in mouse models of diet-induced MASH

**Fig. 5:**
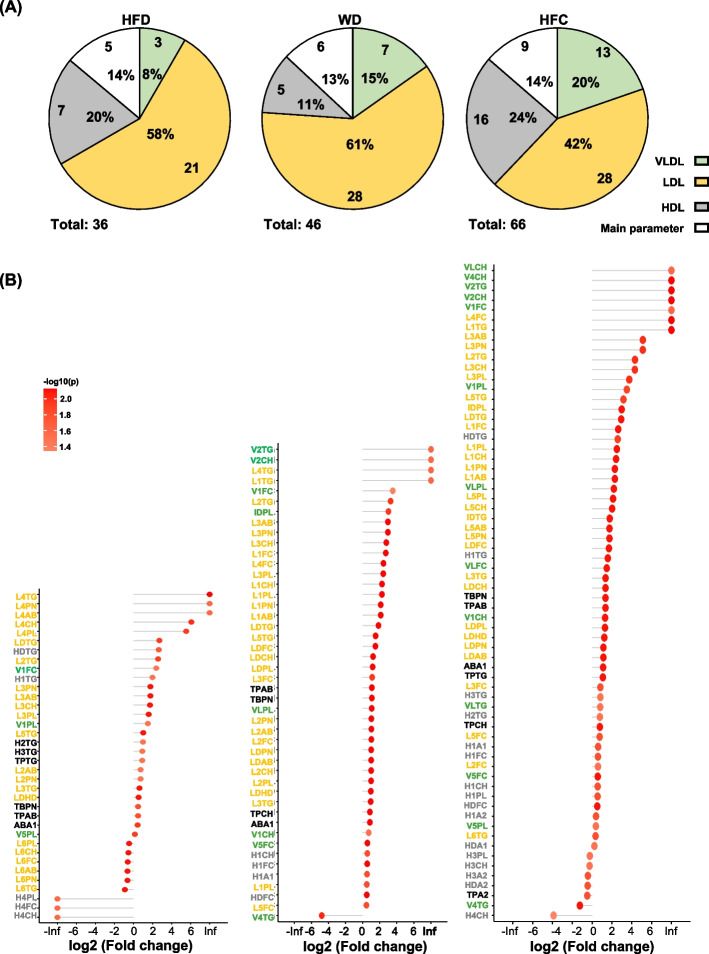
Changes in lipoprotein metabolites with progression of diet-induced metabolic dysfunction-associated steatohepatitis. **A** Pie chart presenting numbers and proportions of lipoprotein metabolites exhibiting gradual changes with diet. **B** Differential expression (32 vs. 16 weeks) of lipoprotein metabolites exhibiting gradual changes with diet. Significant lipoproteins were selected on the basis of a *p* value of < 0.05 and a log2FC value of > 1. Green part represents very low–density lipoprotein, yellow part represents low-density lipoprotein, and grey part represents high-density lipoprotein

The association between phenotypic and metabolomic characteristics indicated that lipoproteins can play a pivotal role in the development of MASLD/MASH. Differential expression analysis was performed to understand the gradual changes in lipoproteins during the progression of diet-induced MASH. The nonnominal method (Wilcoxon rank-sum test) was used for statistical analysis [41]. Significant biomarkers were selected on the basis of a *p* value of < 0.05 and an log2FC value of > 1. Pie charts depicting the results of differential gene expression analysis are presented in Fig. 5A. In the HFD group, significant changes were observed in a total of 36 biomarkers, accounting for 14% of all primary biomarkers. VLDL, LDL, and high-density lipoprotein (HDL) accounted for 8%, 58%, and 20%, respectively, of all biomarkers (Fig. 5A). In the WD group, the primary biomarkers, VLDL, LDL, and HDL accounted for 13%, 15%, 61%, and 11%, respectively, of all biomarkers (Fig. 5A). In the HFC group, the primary biomarkers, VLDL, LDL, and HDL accounted for 14%, 20%, 42%, and 24%, respectively, of all biomarkers (Fig. 5A). The changes in lipoprotein subfractions were ranked and are shown in Fig. 5B. The names, abbreviations, and sizes of all lipoprotein biomarkers are presented in Supplementary Table 3. In the HFD group, the top five metabolites (small LDLs) were L4TG, L4PN, L4AB, L4CH, and L4PL; the expression of these metabolites was considerably upregulated with time. However, the expression of small HDLs, such as H4PL, H4FC, and H4CH, was downregulated. In the WD group, the expression of the following three predominant metabolites was upregulated: the large VLDLs V2TG and V2CH and the large LDL L4TG. The size and number of various lipoproteins exhibited an average distribution; significant increases were particularly observed for LDLs, including for L1TG, L4TG, L2T, L3AB, L3PN, L3CH, L1FC, and L4FC. Regarding HDL, low levels of increases were observed in large HDLs, such as H1CH, H1FC, and H1A1. In the HFC group, considerable changes were noted with time in five VLDLs and two LDLs: the large VLDLs V2TG, V2CH, V1FC, and V4CH and the medium to large LDLs L1TG and L4FC. A significant reduction was noted in one HDL: H4CH. In summary, we observed gradual changes in lipoproteins with the changing severity of MAFSD/MASH. In the HFD, WD, and HFC groups, major changes were observed in small LDLs, large VLDLs and medium LDLs, and large VLDLs and medium to large LDLs, respectively.

**Fig. 6:**
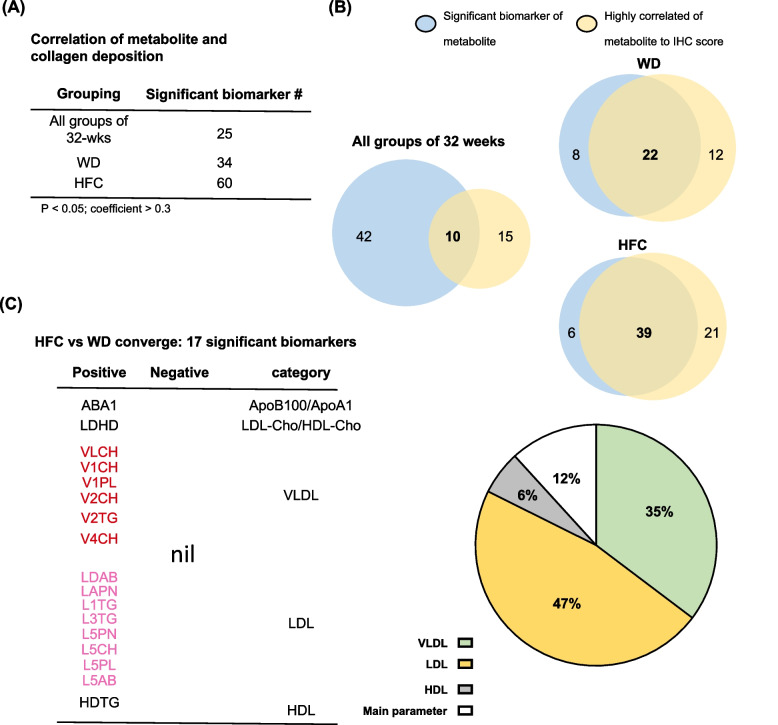
Expression levels of very low–density lipoprotein and low-density lipoprotein are strongly correlated with the severity of metabolic dysfunction-associated steatohepatitis/fibrosis. **A** Correlation between serological metabolites and collagen scores. Significant lipoproteins were selected on the basis of a *p* value of < 0.05 and a correlation coefficient of > 0.3. **B** Integrated analysis of significant metabolites and metabolites strongly associated with fibrosis. Blue indicates significant metabolites, whereas yellow indicates metabolites strongly correlated with fibrosis. **C** Shortened list of 17 significant metabolites identified from the comparison between HFC and WD groups (Table on the left). The pie chart presents the proportions of the 17 significant metabolites

To identify the correlation between metabolites and MASLD/MASH severity, we performed correlation analysis of significant metabolites with collagen deposition (Fig. 6A). A total of 25 significant biomarkers of MASLD were identified in all groups fed for 32 wks. In total, 34 significant biomarkers of MASH were identified in the WD group; moreover, 60 significant biomarkers of severe MASH/fibrosis were identified in the HFC group (Fig. 6A). A comparison of the significant metabolites identified from the experimental groups with the metabolites that were significantly correlated with collagen deposition revealed a high degree of overlapping (Fig. 6B). Pathological analysis (Fig. 2) revealed that although both WD and HFC induced MASH, HFC induced a more severe condition—liver fibrosis. Therefore, we further compared the WD group (mild fibrosis) with the HFC group (severe fibrosis) to explore fibrosis-specific metabolites. A total of 17 metabolites were found to be associated with severe liver fibrosis (Fig. 6C). VLDLs and LDLs accounted for 35% and 47%, respectively, of the aforementioned metabolites; both VLDLs and LDLs were found to be predominant in the liver of mice with severe MASH (Fig. 6C). The 17 markers included the large VLDLs V1CH, V1PL, V2CH, V2TG, and V4CH; the small LDLs L5PN, L5CH, L5PL, and L5AB; and the HDL HDTG (Fig. 6C). Taken together, the results indicate VLDL and LDL are involved in the development of MASLD/MASH and induce severe fibrosis. These findings elucidate both the roles of VLDL and LDL as biomarkers of severe MASH and the pathophysiological changes that occur during the progression of MASH.

### Upregulation of VLDLR expression in mouse models of diet-induced MASH

**Fig. 7:**
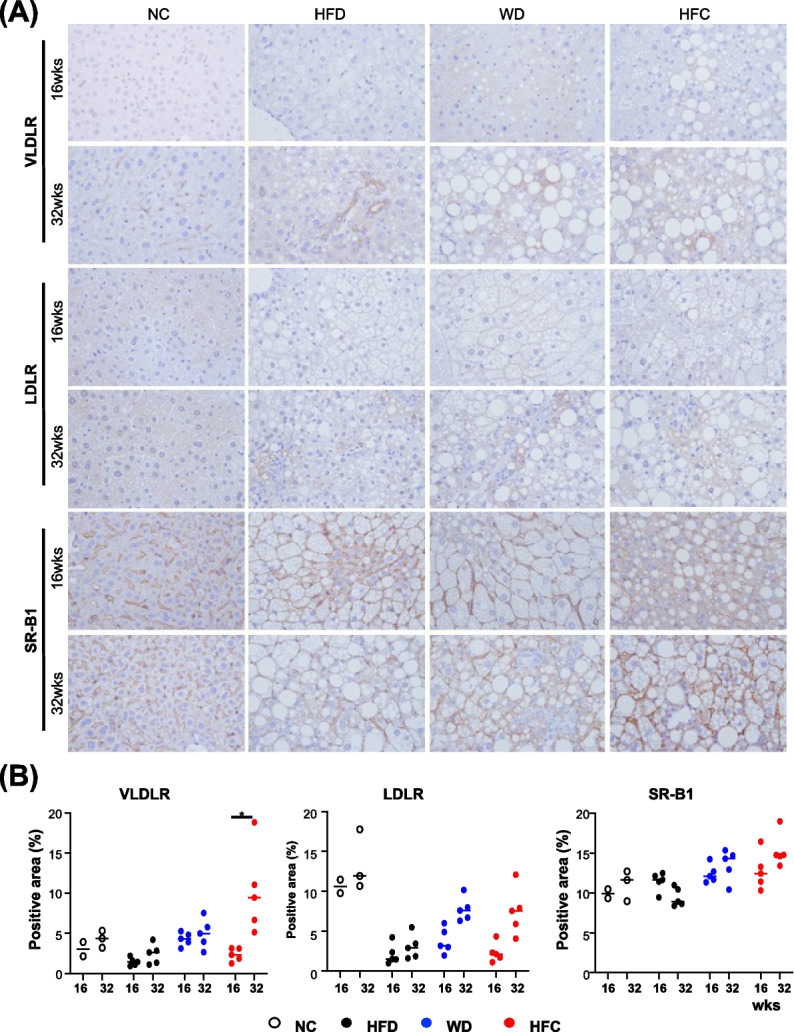
Expression levels of lipoprotein receptors in mouse models of diet-induced MASH. **A** Results of immunohistochemical staining performed to measure the expression levels of very low–density lipoprotein receptor, low-density lipoprotein receptor, and high-density lipoprotein receptor (SR-B1) in the livers of mice with diet-induced MASH. The brown indicates receptor expression. **B** Receptor expression levels were quantified using ImageJ and analyzed using GraphPad Prism (version 8). The white, black, blue, and red dots indicate NC, HFD, WD, and HFC, respectively. Statistical significance: *p < 0.05, compared with NC. NC: normal chow; HFC: high-fat diet; WD: Western diet; HFC: high-fat, high-cholesterol; and MASH: metabolic dysfunction-associated steatohepatitis

The liver is the most prominent contributor of lipoproteins because this organ is responsible for both the production and recycling of lipoproteins [47]. Lipoprotein receptors are crucial for systemic lipid metabolism. The expression of lipoprotein receptors, such as VLDLR, LDL receptor (LDLR), and HDL receptor (SR-B1), in normal organs has been studied in humans and mice [47, 48]. VLDL is believed to be produced only by the liver; VLDLR is expressed in the periphery of but not within the liver [49]. In our study, the expression of both LDL and VLDL was upregulated in mouse models of severe MASH, which prompted us to investigate the receptors of these lipoproteins in diseased liver tissues. The expression and distribution of VLDLR, LDLR, and SR-B1 were evaluated through immunohistochemical analysis (Fig. 7A) and was quantified using ImageJ (Fig. 7B). The expression of VLDLR was not similar between the diet- or feeding time–based groups, with the exception of the HCF group, which was fed for 32 weeks (Fig. 7B). After 16 weeks of feeding, the expression of LDLR was markedly downregulated in the experimental groups compared with that in the control group; nonetheless, the expression was gradually restored in the WD and HFC groups after 32 weeks of feeding (Fig. 7B). Notably, the expression of SR-B1 remained high and did not change with diet (Fig. 7B). In summary, the expression of VLDLR is considerably upregulated in severe liver fibrosis. The findings of increases in the levels of serological VLDL and LDL and the upregulation of VLDLR expression in the severe MASH of this study indicate a feedforward mechanism for lipid deposition.

### VLDLs serve as the biomarkers of liver fibrosis/cirrhosis in humans

**Fig. 8:**
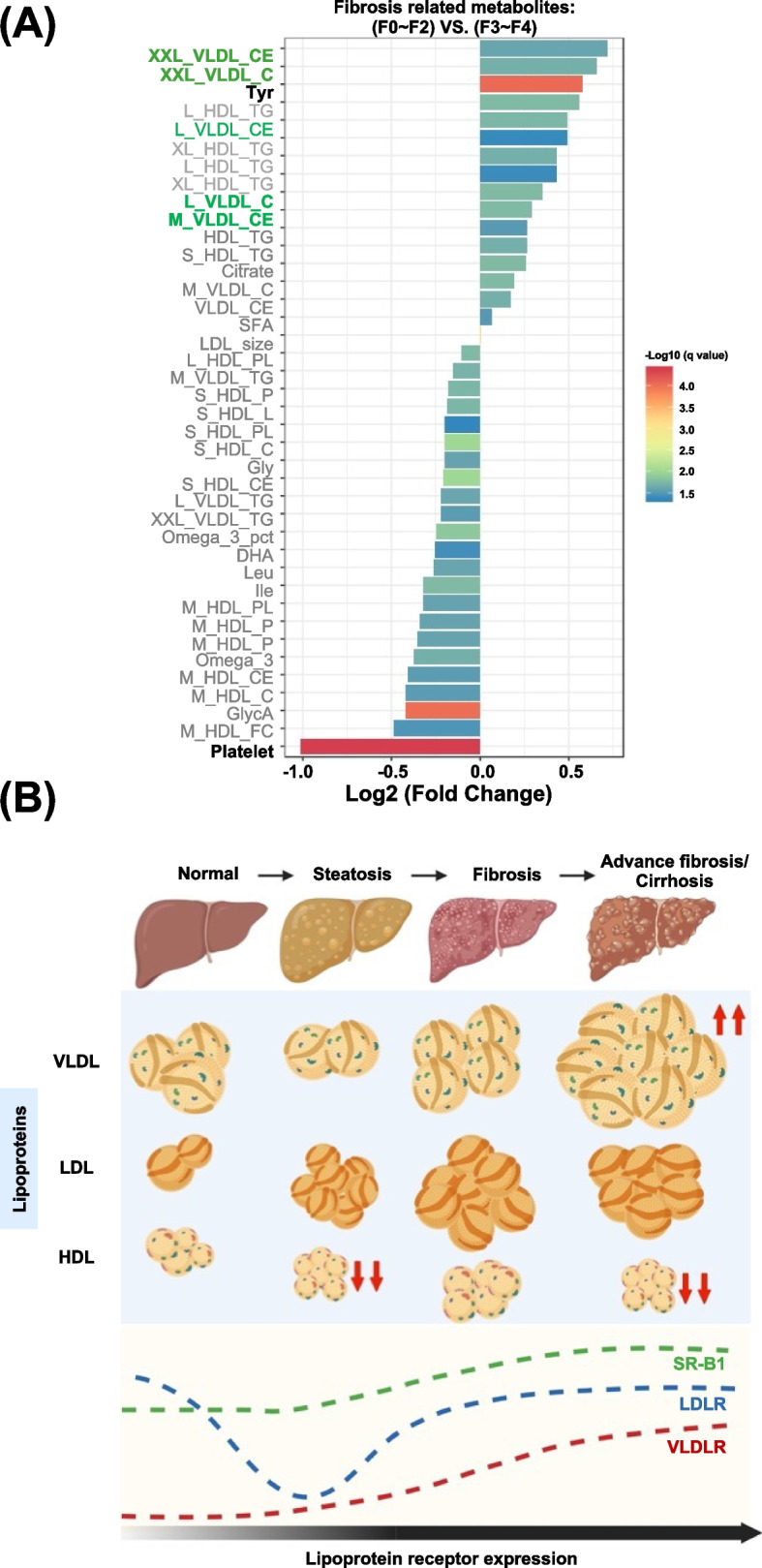
Very low–density lipoprotein and low-density lipoprotein as key biomarkers of fibrosis/cirrhosis in humans. **A** Significant metabolites identified through the differential gene expression analysis of a retrospective cohort of patients with fibrosis/cirrhosis. Log2 fold changes were calculated by comparing the FIB4 scores of patients with advanced disease (F3 or F4) with those of patients with no or mild fibrosis (F0–F2). Significant metabolites associated with cirrhosis. Log2 fold changes were determined by comparing patients with cirrhosis with those without cirrhosis. **B** Changes in lipoproteins during the progression of MASH/fibrosis in mice

The expression of large VLDLs and VLDLR are upregulated in mouse models of diet-induced MASH with severe fibrosis (Figs. 6C and 7). We analyzed the clinical specimens of a retrospective cohort of patients with liver fibrosis/cirrhosis to identify the correlation between serological metabolites and clinical features. The demographic characteristics of our cohort are summarized in Supplementary Table 5. On the basis of their METAVIR scores, the patients were stratified into mild and severe disease groups. A comparison of the metabolome and differential expression of relevant genes were performed. The results revealed significant increases in the levels of the following metabolites (very large VLDLs) in patients with severe fibrosis: XXL_VLDL_CE, XXL_VLDL_C, L_VLDL_CE, and L_VLDL_C (Fig. 8A). Human and experimental (mouse) MASH diseases were similar in terms of the upregulation of the expression of very large VLDLs. In summary, the severity of diet-induced MASH in mouse models can be evaluated to align with clinical diagnostic methods. Metabolomic profiling revealed a likely mechanism of VLDL recycling through VLDLR, which may be involved the pathogenesis of liver fibrosis/cirrhosis.

## Discussion

In this study, three commonly employed experimental diet–induced MASH models were used to evaluate MASH severity by using clinically relevant diagnostic methods. The serological metabolites associated with MASH severity was identified. The roles of the lipoprotein–receptor axes in the pathogenesis of diet-induced MASH in mice were investigated.

### Importance of mouse models of diet-induced MASH in clinical diagnosis

MASH is diagnosed on the basis of histopathological features, such as steatosis, hepatocyte ballooning, and lobular inflammation. Although fibrosis is not a histopathological feature of MASH, it can be used to predict the risk of mortality. The METAVIR scoring system is a commonly used tool for diagnosing fibrosis. The pathogenesis and progression of MASH is complex and involve cellular heterogeneity and alterations in the humoral matrix. Histopathological analysis is important in MASH diagnosis. Rodent models of experimental MASLD/MASH/fibrosis/cirrhosis can be established through diet, genetic modifications, toxin treatment, and a combination of different methods [11, 15, 50]. Few studies have been conducted to systemically compare diet-induced MASLD/MASH models, evaluate their importance in clinical diagnosis, and identify metabolite biomarkers. The present study was conducted to obtain valuable insight into various decompensated liver diseases, such as MASH, fibrosis, and cirrhosis. Inbred rodents are widely used for studying MASLD/MASH from a genetic perspective [51]. The present study was conducted using C57BL/6 mice, which are commonly used in transgenic animal studies.

### Importance of noninvasive metabolomic tools in MASH diagnosis

The METAVIR scoring system is an invasive tool that involves liver biopsy, which involves the risks of major vein rupture and internal bleeding. Serological biomarkers, such as FIB4, can serve as noninvasive tools for disease diagnosis. However, the precision of clinical diagnosis performed on the basis of FIB4 is low (receiver operating characteristic curve score, approximately 70%) [52, 53]. In our study, high-throughput metabolomic profiling was performed with NMR spectroscopy; the results revealed a correlation between the phenotypic and metabolomic characteristics of mouse models of MASH. In addition, a novel biomarker of steatohepatitis was identified.

Regarding the relevance of experimental MASH to human MASH, elevated levels of VLDL, VLDL-cholesterol, and LDL-cholesterol can serve as the biomarkers of the progression of hepatic steatosis to MASH (Fig. 8A). High serum levels of total lipid and cholesterol (VLDL and LDL) are associated with intrahepatic cholesterol accumulation and hepatocyte injury in MASH [54, 55]. The similarity between experimental diet-induced MASH and human MASH in terms of lipoprotein metabolites indicated lipoprotein analysis may be valuable for clinical diagnosis. Therefore, the significance of our study lies in its identification of an association of large VLDLs and LDLs with the progression of MASH in animal models and patients (Fig. 8B).

Most studies conducted using animal models of MASH have explored lipid, glucose, and protein metabolites in the liver. Our findings reveal that the levels of lipoproteins increased with the severity of MASH. Through NMR spectroscopy–based metabolomic profiling, both VLDL and LDL were simultaneously explored in mice and humans. The consistency between the experimental MASH and human MASH in terms of metabolite biomarkers indicated that similarities were present in the pathophysiological alternations between the mouse and human, and that NMR spectroscopy–based metabolomic profiling can be valuable for research and clinical diagnosis.

### Lipoproteins as the predominant biomarkers of diet-induced MASH in mice

Serum-insoluble lipids circulate in the bloodstream as lipoproteins, which are macromolecular complexes of free cholesterol, cholesterol esters, triglycerides, phospholipids, and apolipoproteins [56]. The liver is the primary site for the synthesis of LDL (16–30 nm) and VLDL (30–80 nm), which carry lipids and ApoB100 to tissue. An essential lipoprotein for the collection and transportation of extra serum cholesterol is HDL (8–16 nm; ApoA). Structurally, VLDL comprises a triglyceride-enriched core surrounded by a monolayer of phospholipids and incorporated proteins (e.g., ApoB-100), which facilitates the delivery and uptake of VLDL. Notably, most triglycerides incorporated into VLDL are derived from exogenous lipids and not synthesized through de novo lipogenesis [57, 58]. The impaired synthesis of ApoB-100 in patients with MASH may be associated with increased free fatty acid level, disrupted redox balance, hyperinsulinemia, and reduced gene expression, all of which hinder ApoB-100 synthesis and VLDL assembly, thus resulting in intrahepatic lipid accumulation [59]. Additionally, VLDL particles can be converted into LDL particles through hydrolyzation of triglycerides by LPL in the bloodstream [56].

In patients with MASLD, the expression of VLDL in the liver is upregulated, leading to increased levels of triglycerides. In addition, the clearance of LDL is reduced, which accelerates the development of atherosclerosis and cardiovascular disease. Excessive lipid storage in the liver promotes the secretion of VLDL and thus dyslipidemia [60]. Furthermore, an increase in the level of oxidized LDL occurs, which induces systemic inflammation [60].

The increase in the mean size of VLDL in patients with MASH and the reduction in the level of small VLDL in patients with liver fibrosis reflect changes in the number and state of hepatocytes resulting from such diseases [61]. Hepatocytes with increased levels of intracellular lipid can serve as the source of large VLDLs. MASH driven by insulin resistance and an increase in the intrahepatic lipid pool may increase the numbers of large VLDLs and the mean size of VLDLs [62–64].

### Upregulation of VLDL/VLDLR expression indicates a positive feedforward mechanism for hepatic lipid accumulation

In adipose tissues, VLDLR is regulated by peroxisome proliferator–activated receptor [65]. Free cholesterol and fatty acid can promote stress response, inflammation, apoptosis, and fibrosis in the liver [66]. Large VLDLs induce the accumulation of triglycerides in macrophages and exhibit a higher affinity for VLDLR binding than do small VLDLs. Disease states may influence VLDL properties. Hepatic secretion of VLDL is impaired in patients with *ApoB* mutations, which often leads to fatty liver disease because of the excessive intrahepatic accumulation of fat. The extent of steatosis is associated with the size and number of VLDLs in the patient population [67]. Patients with hepatic steatosis and insulin resistance have increased levels of circulating ApoC-III, which is a strong inhibitor of LPL. After the LPL-mediated hydrolysis of triglycerides, lipoprotein remnants are removed through receptor-mediated pathways, primarily those operated in the liver. Obesity and insulin resistance contribute to reduced LDL clearance by reducing the activities of LDLR and LDLR-related protein 1, among others [68]. The intrahepatic accumulation of LDL due to reduced receptor-mediated uptake may directly inhibit LPL, resulting in a feedforward mechanism that drives lipid deposition and MASH development [69–71].

The dysregulation of lipid homeostasis in hepatocytes leads to the generation of toxic lipids that impair organelle functions, promoting inflammation, hepatocellular damage, and apoptosis [69]. In patients with MASH, the uptake of circulating lipids, particularly free fatty acids and lipoproteins, by the liver is higher [72]. Because the hepatic secretion of lipoprotein is higher in patients with MASLD, the deposition of fat in hepatocytes disrupts lipid homeostasis in these cells [72]. Further studies are required to evaluate the quantity and quality of changes in lipid metabolites during the pathogenesis of MASH.

## Conclusions

Our findings provide key insight into the pathophysiology and serological metabolomics of experimental diet-induced MASH in relation to human MASH. The finding of an upregulation of lipoprotein expression indicates a feedforward mechanism underlies MASH development, and this mechanism may be targeted for the development of noninvasive diagnostic strategies.

### Supplementary Information

**Additional file 1:** **Supplemental Fig. 1.** Trends of total metabolites in mouse models of diet-induced metabolic dysfunction-associated steatohepatitis. (A) Plot generated through principal component analysis (PCA) of the total metabolites of mice fed with NC, HFD, WD, or HFC (16 and 32 wks). PC1: principal component 1; PC2: principal component 2. Each point represents the metabolite profile of a biological replicate. (B) Heatmap exhibiting prominent differences in total metabolites patterns among NC, 16-wk experimental diet, and 32-wk experimental diet. NC: normal chow; HFD: high-fat diet; WD: Western diet; and HFC: high-fat, high-cholesterol diet. **Supplemental Table 1.** List of real-time PCR primer. **Supplemental Table 2.** List of all metabolite in human. **Supplemental Table 3.** List of all lipoprotein subclass of mouse. **Supplemental Table 4.** List of small metabolites of mouse. **Supplemental Table 5.** Baseline characteristics of the study cohort.

## Data Availability

All data generated or analysed during this study are included in this published article and its supplementary information files.
