# Supplementary material for: Phenotypic and metabolomic characteristics of mouse models of metabolic associated steatohepatitis
Source: Biomark Res. 2024 Jan 9;12:6. doi: 10.1186/s40364-023-00555-9 (PMC10777576; doi:10.1186/s40364-023-00555-9)
Supplement: Supplementary file 1 — Additional file 1: Supplemental Fig. 1. Trends of total metabolites in mouse models of diet-induced metabolic dysfunction-associated steatohepatitis. (A) Plot generated through principal component analysis (PCA) of the total metabolites of mice fed with NC, HFD, WD, or HFC (16 and 32 wks). PC1: principal component 1; PC2: principal component 2. Each point represents the metabolite profile of a biological replicate. (B) Heatmap exhibiting prominent differences in total metabolites patterns among NC, 16-wk experimental diet, and 32-wk experimental diet. NC: normal chow; HFD: high-fat diet; WD: Western diet; and HFC: high-fat, high-cholesterol diet. Supplemental Table 1. List of real-time PCR primer. Supplemental Table 2. List of all metabolite in human. Supplemental Table 3. List of all lipoprotein subclass of mouse. Supplemental Table 4. List of small metabolites of mouse. Supplemental Table 5. Baseline characteristics of the study cohort. [file 40364_2023_555_MOESM1_ESM.docx]

**Supplemental information**

**Supplemental Fig. 1**

**
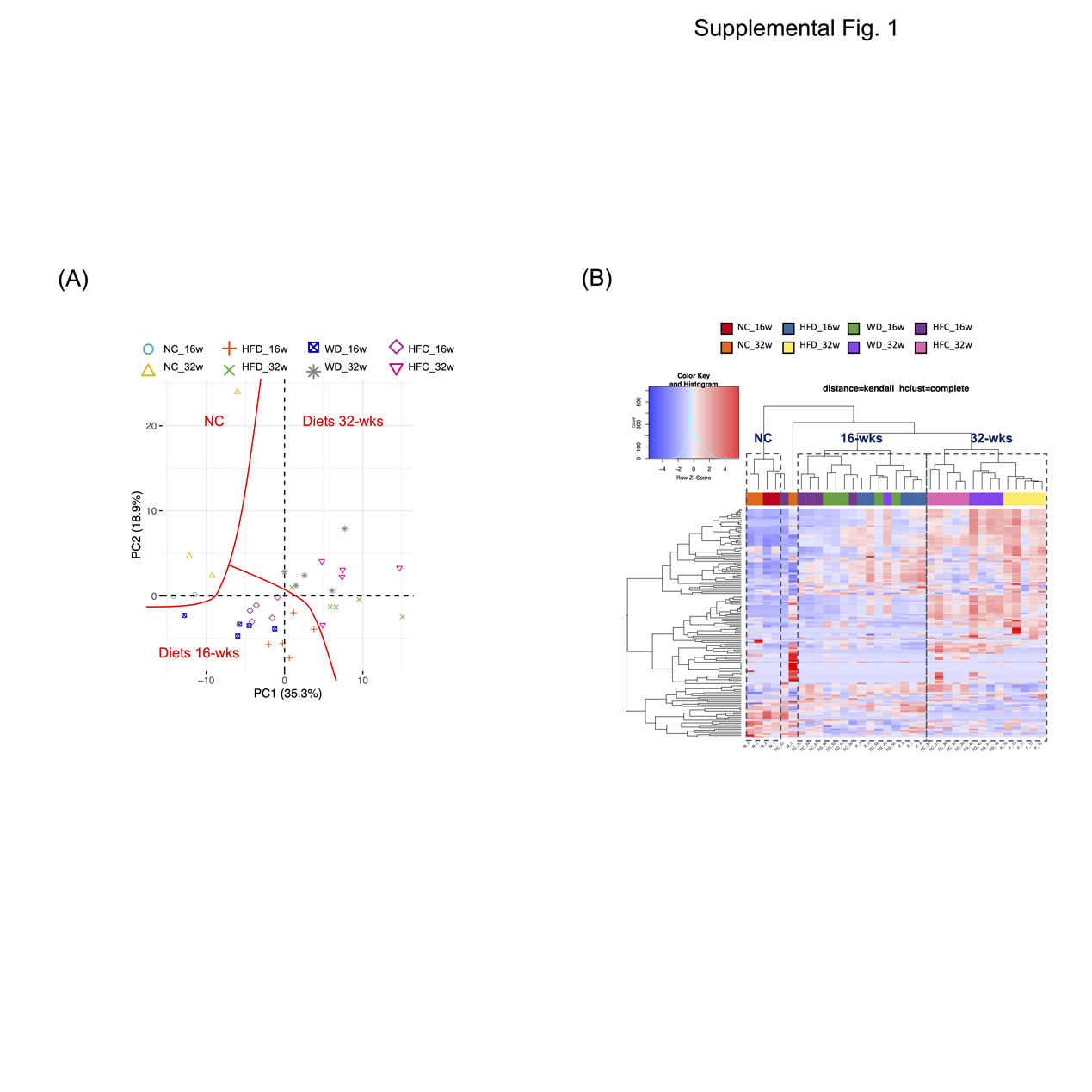
**

**Supplemental Fig. 1. Trends of total metabolites in mouse models of diet-induced metabolic dysfunction-associated steatohepatitis.**

(**A**) Plot generated through principal component analysis (PCA) of the total metabolites of mice fed with NC, HFD, WD, or HFC (16 and 32 wks). PC1: principal component 1; PC2: principal component 2. Each point represents the metabolite profile of a biological replicate. (**B**) Heatmap exhibiting prominent differences in total metabolites patterns among NC, 16-wk experimental diet, and 32-wk experimental diet. NC: normal chow; HFD: high-fat diet; WD: Western diet; and HFC: high-fat, high-cholesterol diet.

**Supplemental Table. 1 List of real-time PCR primer**

| **Primer** | | **Sequence (5′–3′)** |
| --- | --- | --- |
| *β-actin* | Forward | TCACCCACACTGTGCCCATCTACGA |
|  | Reverse | CAGCGGAACCGCTCATTGCCAATGG |
| *Collagen I* | Forward | CCTCAGGGTATTGCTGGACAAC |
|  | Reverse | CAGAAGGACCTTGTTTGCCAGG |
| *a-SMA* | Forward | TGCTGACAGAGGCACCACTGAA |
|  | Reverse | CAGTTGTACGTCCAGAGGCATAG |

**Supplemental Table. 2 List of all metabolite in human**

**Supplemental Table. 3. List of all lipoprotein subclass of mouse**

**Supplemental Table. 4. List of small metabolites of mouse**

**Supplemental Table 5. Baseline characteristics of the study cohort**

| **Demographics** | **All (n = 80)** | | | No cirrhosis  (n = 46) | Cirrhosis (n = 34) | | p-value |  |  |
| --- | --- | --- | --- | --- | --- | --- | --- | --- | --- |
| Age, years | | 60.8 ± 9.1 | 62.1 ± 9.3 | | | 59.1 ± 8.6 | 0.15 |  |  |
| Male (%) | | 61 (76.2) | 35 (76.1) | | | 26 (76.5) | 1 |  |  |
| BMI | | 24.5 ± 3.8 | 25 ± 3.6 | | | 23.8 ± 4 | 0.2 |  |  |
| HBsAg^+^(%) | | 36 (45) | 20 (43.5) | | | 16 (47.1) | 0.82 |  |  |
| HCVAb^+^ (%) | | 32 (40) | 16 (34.8) | | | 16 (47.1) | 0.36 |  |  |
| Type 2 diabetes (%) | | 28 (35) | 10 (21.7) | | | 18 (52.9) | **0.005** |  |  |
| **Sero-biochemistry parameters** | | | | | | | |  |  |
| Albumin (g/dL) | | 3.6 ± 0.6 | | 3.8 ± 0.5 | | 3.4 ± 0.6 | **0.001** |  |  |
| Total bilirubin (mg/dL) | | 1.5 ± 1.5 | | 1 ± 0.6 | | 2.2 ± 2.1 | **0.003** |  |  |
| Creatinine (mg/dL) | | 1.4 ± 2.8 | | 1.5 ± 3.6 | | 1.2 ± 0.8 | 0.54 | | |
| Platelet (10^3^/µL) | | 162.4 ± 96.9 | | 207.1 ± 102.4 | | 102 ± 40.2 | **<0.0001** |  |  |
| **Tumor related** | |  | |  | |  |  |  |  |
| Tumor size (cm) | | 5.1 ± 4.9 | | 6.1 ± 6 | | 3.7 ± 2.4 | **0.017** |  |  |
| AFP > 400 (%) | | 15 (18.8) | | 10 (21.7) | | 5 (14.7) | 0.57 |  |  |
| Stage III + Stage IV (%) | | 23 (28.6) | | 11 (32.4) | | 12 (26.1) | 0.62 |  |  |
| Microscopic venous invasion (%) | | 21 (26.2) | | 13 (28.3) | | 8 (23.5) | 0.8 |  |  |
| Macroscopic venous invasion (%) | | 8 (10) | | 4 (8.7) | | 4 (11.8) | 0.72 |  |  |
| Lymph node involvement (%) | | 12 (15) | | 7 (15.2) | | 5 (14.7) | 1 |  |  |
| Values are the mean ± SD, unless otherwise noted as n (%). P-values were calculated by comparing patients with and without cirrhosis using a two-tailed t-test for continuous variables or Fisher’s exact test for categorical variables. Red bold indicated p-values<0.05. BMI, body mass index; HBsAg, hepatitis B virus surface antigen; HCVAb, hepatitis C virus antibody; AST, aspartate aminotransferase; ALT, alanine aminotransferase; AFP, α-fetoprotein | | | | | | | | |  |

**Material & Methods**

**Real-time quantitative polymerase chain reaction**

Total RNA was extracted from the liver tissues of the mice by using TRIzol (Cat: T9424, Sigma-Aldrich, St. Louis, Missouri, USA ) according to the manufacturer’s instructions.(*1*) The obtained RNA (5 μg) was reverse-transcribed using the PrimeScript RT Reagent Kit (Cat: RR037A; TaKaRa, Tokyo, Japan) according to the manufacturer’s instructions.(*2*)Quantitative polymerase chain reaction (PCR) was performed using the KAPA SYBR FAST qPCR Master Mix (KM4100; KAPA Biosystems) with specific primers (Supplementary Table 1); for this, the Azure Cielo Real-Time PCR System (Azure Biosystems, Dublin, CA, USA) was used. Gene expression levels were normalized against the expression level of actin, and the relative changes in gene expression were quantified using the 2^−ΔΔCt^ method.

**Detection of hyaluronan through sandwich enzyme-linked immunosorbent assay**

The presence of hyaluronan in the serum samples of the experimental mice was detected through sandwich enzyme-linked immunosorbent assay (ELISA). All blood samples were centrifuged at 1000 ×*g* for 15 min at 4°C; the obtained plasma samples were subjected to sandwich ELISA, which was performed using Quantikine ELISA Kits (Cat#: DHYAL0; R&D System, Minneapolis, MN, USA) according to the manufacturer’s instructions. In brief, all reagents, working standards, and samples were prepared as directed. To each well, 50 μL of Assay Diluent RD1-14 was added followed by 50 μL of the standard, control, or sample. The ELISA plate was incubated on a horizontal orbital shaker (70 rpm) for 2 h at 25°C. After incubation, each well was aspirated and washed five times with 400 μL Wash Buffer. Next, 100 μL of hyaluronan conjugate was added to each well. The plate was covered with a clean adhesive strip and incubated on the shaker for 2 h at room temperature. After incubation, the wells were aspirated and washed as indicated. Subsequently, 100 μL of substrate solution was added to each well. The plate was incubated in the dark for 30 min at room temperature. After incubation, 100 μL of stop solution was added to each well. Within 30 min of the addition of the stop solution, absorbance was measured at 450 and 570 nm by using a microplate reader; for background correction, the readings obtained at 570 nm were subtracted from those obtained at 450 nm.

**NMR** **spectroscopy–based metabolomic profiling**

A retrospective cohort of patients with cirrhosis was analyzed. Serum samples were collected from 80 patients with HCC and subjected to NMR spectroscopy–based metabolomic profiling by using the Nightingale Health platform (Helsinki, Finland).(*3*) This platform facilitates the simultaneous detection of 151 serum biomarkers and provides a comprehensive spectrum of metabolites. The biomarkers include lipid metabolites, such as cholesterol, triglycerides, various fatty acids, apolipoprotein (Apo)A1, and ApoB; amino acid~~s~~; glycolysis-related metabolite~~s~~; ketone bodies; creatinine; albumin; and glycoprotein acetyls. The size, subclass distribution, and loading lipids of lipoprotein metabolites can also be analyzed using this platform. Details regarding the observed serological metabolites, including their abbreviations and units, are presented in Supplementary Table 2.

For metabolomic profiling, mouse plasma samples were assessed through NMR spectroscopy (Ascend 600C) on the Burker high-throughput metabolomics platform. The standardized platform included Bruker IVDr Lipoprotein Subclass Analysis (model version: PL-5009-01/001) and the automated quantification of small metabolites (model version: Quant-PS 2.0.0). Details regarding the metabolites, including their abbreviations and units, are presented in Supplementary Tables 3 and 4.

**Retrospective analysis of patients with fibrosis or cirrhosis**

A total of 113 patients who received a confirmed diagnosis of HCC between 2009 and 2013 at China Medical University Hospital were retrospectively included in this study. Patients with missing liver biopsy or key hematological data; those with blood samples unsuitable for metabolite quantification; those aged <18 or >80 years; those receiving long-term drug therapy; and those with advanced cancer metastasis, HIV infection, autoimmune disease, or other liver-related comorbidities (e.g., Wilson’s disease, haemochromatosis, alpha-1 antitrypsin deficiency, lupoid hepatitis, and cholestatic or vascular liver disorders) were excluded from this study. Finally, 80 patients were included in the analysis. The patients’ demographic and medical data were recorded by trained research assistants. The effects of the following covariates were adjusted in the models used for statistical analysis: age (years), sex (male or female), body mass index (kg/m^2^), smoking status (yes [smoked for at least 1 year] or no), hypertension (blood pressure of >140/90 mmHg or previous physician diagnosis), type 2 diabetes (fasting glucose level of >126 mg/dL or previous physician diagnosis), hepatitis B surface antigen (yes or no), hepatitis C antibody (yes or no), Child–Pugh score (A+B or C), ascites (yes or no), hepatic steatosis (yes or no), tumor size (cm), cancer stage (I+II or III+IV), microscopic venous invasion (yes or no), macroscopic venous invasion (yes or no), lymph node involvement (yes or no), and capsule (yes or no) and satellite nodules (yes or no). This study was approved by the Ethics Committee of China Medical University. Written informed consent was obtained from all patients.

**Bioinformatic analyses**

For metabolite analysis, all data were subjected to normality tests (Shapiro–Wilks and Anderson–Darling tests)(*4*) and Q–Q plot analysis.(*4*) Among the metabolites, >50% were abnormally distributed; therefore, subsequent analyses were performed using nonparametric tests.(*5*) The metabolites were divided into three groups: lipoproteins and small metabolites, lipoprotein metabolites, and small metabolites.

The metabolite groups were subjected to principal component analysis (PCA).(*5, 6*) Hierarchical clustering heatmaps were independently constructed using R package gplots.(*7, 8*) Before analysis, each feature was scaled to have a mean value of 0 and a standard deviation value of 1.

Multiple comparisons were performed using the Kruskal–Wallis test(*9-11*) in different groups of diets. Statistical significance was set at *p* < 0.05. To analyze the differentially expressed metabolites, the Wilcoxon rank-sum test(*12*) was performed, with fold changes and adjusted *p* values calculated for between-group comparisons. Metabolites were considered to have significance when the corresponding *p* values were <0.05 and log2 fold changes were greater than or equal to 1 or less than or equal to −1. Spearman correlation analysis(*13*) was performed to investigate the correlation between collagen scores and metabolites. A *p* value of <0.05 and a correlation coefficient of >0.3 indicated statistical significance.

For the cohort of patients with cirrhosis, between-group comparisons of metabolites were performed using a two-tailed Student’s *t* test. The resulting *p* values were adjusted for multiple comparisons by using the Benjamini–Hochberg correction method.(*14, 15*) Statistical significance was set at a q value of <0.05. Significant metabolites were selected to construct a Pearson correlation matrix,(*16*) which was subsequently subjected to hierarchical cluster analysis, in which the Euclidean distance(*17*) was measured using the average method. All statistical analyses were performed using R (version 4.1.0).

**Reference**

1. Tri Reagent for RNA Isolation from tissues cells. *Sigma-Aldrich Co. LLC.*, (2021).

2. PrimeScriptTM RT reagent Kit (Perfect Real Time). *Takara Bio Inc*, (2022).

3. P. Wurtz *et al.*, Quantitative Serum Nuclear Magnetic Resonance Metabolomics in Large-Scale Epidemiology: A Primer on -Omic Technologies. *Am J Epidemiol* **186**, 1084-1096 (2017).

4. B. W. Yap, C. H. Sim, Comparisons of various types of normality tests. *J Stat Comput Sim* **81**, 2141-2155 (2011).

5. Y. Chen, E. M. Li, L. Y. Xu, Guide to Metabolomics Analysis: A Bioinformatics Workflow. *Metabolites* **12**, (2022).

6. K. K. Pasikanti *et al.*, Noninvasive urinary metabonomic diagnosis of human bladder cancer. *J Proteome Res* **9**, 2988-2995 (2010).

7. P. H. Benton *et al.*, An Interactive Cluster Heat Map to Visualize and Explore Multidimensional Metabolomic Data. *Metabolomics* **11**, 1029-1034 (2015).

8. H. H. Draisma *et al.*, Hierarchical clustering analysis of blood plasma lipidomics profiles from mono- and dizygotic twin families. *Eur J Hum Genet* **21**, 95-101 (2013).

9. M. Vinaixa *et al.*, A Guideline to Univariate Statistical Analysis for LC/MS-Based Untargeted Metabolomics-Derived Data. *Metabolites* **2**, 775-795 (2012).

10. Y. Y. Pan, Y. C. Chen, W. C. W. Chang, M. C. Ma, P. C. Liao, Visualization of statistically processed LC-MS-based metabolomics data for identifying significant features in a multiple-group comparison. *Chemometr Intell Lab* **210**, (2021).

11. N. M. Razali, Y. B. Wah. (2011).

12. H. Y. Kim, Statistical notes for clinical researchers: Nonparametric statistical methods: 2. Nonparametric methods for comparing three or more groups and repeated measures. *Restor Dent Endod* **39**, 329-332 (2014).

13. W. W. Wang *et al.*, Altered fecal microbiome and metabolome in adult patients with non-cystic fibrosis bronchiectasis. *Respir Res* **23**, 317 (2022).

14. S. Y. Chen, Z. Feng, X. Yi, A general introduction to adjustment for multiple comparisons. *J Thorac Dis* **9**, 1725-1729 (2017).

15. Y. Benjamini, Y. Hochberg, Controlling the false discovery rate: a practical and powerful approach to multiple testing. *Journal of the Royal statistical society: series B (Methodological)* **57**, 289-300 (1995).

16. R. Steuer, Review: on the analysis and interpretation of correlations in metabolomic data. *Brief Bioinform* **7**, 151-158 (2006).

17. Z. Qi, E. O. Voit, Strategies for Comparing Metabolic Profiles: Implications for the Inference of Biochemical Mechanisms from Metabolomics Data. *IEEE/ACM Trans Comput Biol Bioinform* **14**, 1434-1445 (2017).
